# Nested Cross-Validation Reveals Performance Inflation in MRI Radiomics for Early Mortality Prediction in IDH-Wildtype Glioblastoma

**DOI:** 10.3390/cancers18162595

**Published:** 2026-08-12

**Authors:** Lucas I. Becker, Nicolas Noel Neidert, Roberto Doria-Medina, Manou Overstijns, Maryam Wendeberg, Urs Würtemberger, Horst Urbach

**Affiliations:** 1 Department of Neuroradiology, University Medical Centre Freiburg, 79100 Freiburg, Germany; maryam.mirkoei@uniklinik-freiburg.de (M.W.); horst.urbach@uniklinik-freiburg.de (H.U.); 2 Department of Neurosurgery, University Medical Centre Freiburg, 79100 Freiburg, Germany; nicolas.neidert@uniklinik-freiburg.de (N.N.N.); roberto.doria@uniklinik-freiburg.de (R.D.-M.); manou.overstijns@uniklinik-freiburg.de (M.O.); 3 Raya Diagnostics, 80802 Munich, Germany

**Keywords:** glioblastoma, radiomics, nested cross-validation, early mortality, data leakage, MRI, prognostic biomarkers, machine learning

## Abstract

**Background**: Glioblastoma (GBM) carries a median survival of 15 months. Approximately 16–27% of patients die within six months despite standard therapy. Radiomic MRI features have been proposed as prognostic biomarkers, yet many published studies employ standard cross-validation (CV) with feature selection on the full dataset, introducing data leakage. **Methods**: Sixty patients with IDH-wildtype GBM from the publicly available UCSF-PDGM dataset (single-center, GE Discovery MR750, 3 T; 2015–2021) with BraTS 2021 segmentation masks were analyzed. One thousand two hundred eighty-four (1284) IBSI-compliant radiomic features (7 feature classes × 4 MRI sequences × 3 tumor subregions) were extracted using PyRadiomics. After preprocessing (variance filter, correlation filter |r| > 0.95), approximately 120 features remained per fold. Feature selection was performed strictly within each training fold of a nested 5-fold cross-validation framework (5-fold × 3 repeats = 15 outer folds). **Results**: For 1-year mortality, radiomics AUC dropped from 0.816 (standard CV) to 0.593 (nested CV; ΔAUC = −0.223, 27% inflation), while clinical models remained stable (0.708 vs. 0.705). For early mortality (≤180 days, *n* = 16 events), standard CV inflated radiomics AUC to 0.888, whereas nested CV yielded 0.815 (ΔAUC = −0.073, 8% inflation). In feature stability analysis, whole-tumor surface area (13/15 folds) and mesh volume (10/15 folds) showed the highest cross-endpoint stability; nine of 13 exploratory OS-associated features were never selected for early mortality classification. Extent of resection (HR = 0.44, *p* = 0.009) and age (HR = 1.03, *p* = 0.038) were independently associated with overall survival; tumor surface area remained independently associated with survival (HR = 1.38, *p* = 0.021). **Conclusions**: Nested cross-validation revealed substantial performance inflation in standard radiomics pipelines. These descriptive inflation estimates are specific to this dataset and pipeline configuration and should not be generalized as universal parameters for radiomics.

## Simple Summary

Glioblastoma is the most aggressive brain tumor. About one in six patients die within six months despite multimodal therapy. We tested whether computer-extracted MRI features (radiomics) can identify these patients early. We found that a common validation strategy used in radiomics studies overestimates prediction accuracy by up to 27% due to a statistical error called data leakage. When we applied a strict validation method that prevents this error, radiomics still predicted early mortality moderately well (AUC = 0.815). The most stable and interpretable individual features were shape-based, particularly whole-tumor surface area. Our findings suggest that many published radiomics results are overly optimistic, but that a meaningful predictive signal—particularly from tumor morphology—persists under rigorous validation.

## 1. Introduction

Glioblastoma (GBM), IDH-wildtype, CNS WHO grade 4, is the most common malignant primary brain tumor in adults [1]. Despite maximal safe resection followed by radiotherapy and temozolomide according to the Stupp protocol, median overall survival remains approximately 15 months [1,2]. Approximately 16–27% of patients experience early mortality, defined as death within six months of diagnosis [3,4]. Reliable identification of these high-risk patients before or shortly after treatment initiation remains a challenge.

Established prognostic factors include age, extent of resection (EOR), O6-methylguanine-DNA methyltransferase (MGMT) promoter methylation status, and Karnofsky Performance Status [5,6]. However, these variables provide only modest prognostic discrimination, and substantial heterogeneity in survival outcomes persists among patients with similar clinical profiles [7].

Radiomics—the extraction of quantitative imaging features from routine clinical images—has emerged as a promising approach for prognostic biomarker discovery in neuro-oncology [8,9]. Radiomic texture and shape features capture tumor heterogeneity that is often imperceptible to visual inspection and may reflect underlying tumor biology [10,11]. Several studies have reported high discriminative performance for survival prediction in GBM, with AUC values exceeding 0.80 [12,13,14,15].

However, the radiomics field faces a critical methodological challenge. Most published studies perform feature selection on the entire dataset before cross-validation splitting. This practice allows information from the test set to influence feature selection, constituting data leakage [16,17]. The resulting performance estimates are biased upward and unlikely to generalize to independent cohorts. This methodological flaw contributes to the well-documented reproducibility crisis in radiomics research [18,19].

Nested cross-validation addresses this problem by performing all data-driven steps—including feature selection and model selection—exclusively within each training fold of the outer loop [16,20]. Test data never influence model development. This approach yields leakage-controlled performance estimates but is rarely employed in published radiomics studies.

The objectives of this study were:

(1) to quantify the magnitude of performance inflation caused by standard cross-validation in GBM radiomics;

(2) to evaluate radiomic features for predicting early mortality (≤6 months) under strict nested cross-validation;

(3) to identify FDR-corrected radiomic features associated with overall survival;

(4) to assess the independent association of radiomics with overall survival beyond clinical variables using Cox regression.

## 2. Materials and Methods

### 2.1. Study Population

This retrospective study analyzed patients from the UCSF Preoperative Diffuse Glioma MRI (UCSF-PDGM) dataset, version 3 [21]. All imaging was acquired at a single center on a GE Discovery MR750 3-Tesla scanner (8-channel head coil; GE Healthcare, Milwaukee, WI, USA) between 2015 and 2021. Tumor segmentation masks were obtained from the BraTS 2021 Challenge dataset [22,23]. The 2016 WHO classification distinguished glioblastoma, IDH-wildtype from glioblastoma, IDH-mutant [24]; under the 2021 WHO classification, the latter is classified as astrocytoma, IDH-mutant, CNS WHO grade 4 [6].

Inclusion criteria were: (1) histopathologically confirmed IDH-wildtype glioblastoma per WHO 2021 classification [6]; (2) complete multiparametric MRI, including T1-weighted, T1-weighted contrast-enhanced (T1ce), T2-weighted, and FLAIR sequences; (3) available expert-validated tumor segmentation; (4) documented overall survival data with minimum follow-up of 180 days or death before 180 days; and (5) known MGMT promoter methylation status.

Exclusion criteria were: (1) IDH-mutant astrocytoma, CNS WHO grade 4 (*n* = 6, reclassified per WHO 2021); (2) incomplete imaging data; and (3) missing survival information.

The final cohort comprised 60 patients. All patients had follow-up ≥180 days or documented death before 180 days; for the 1-year endpoint, patients with follow-up <365 days who were alive at last contact (*n* = 0) were excluded. Cohort characteristics are summarized in Table 1.

**Figure 1:**
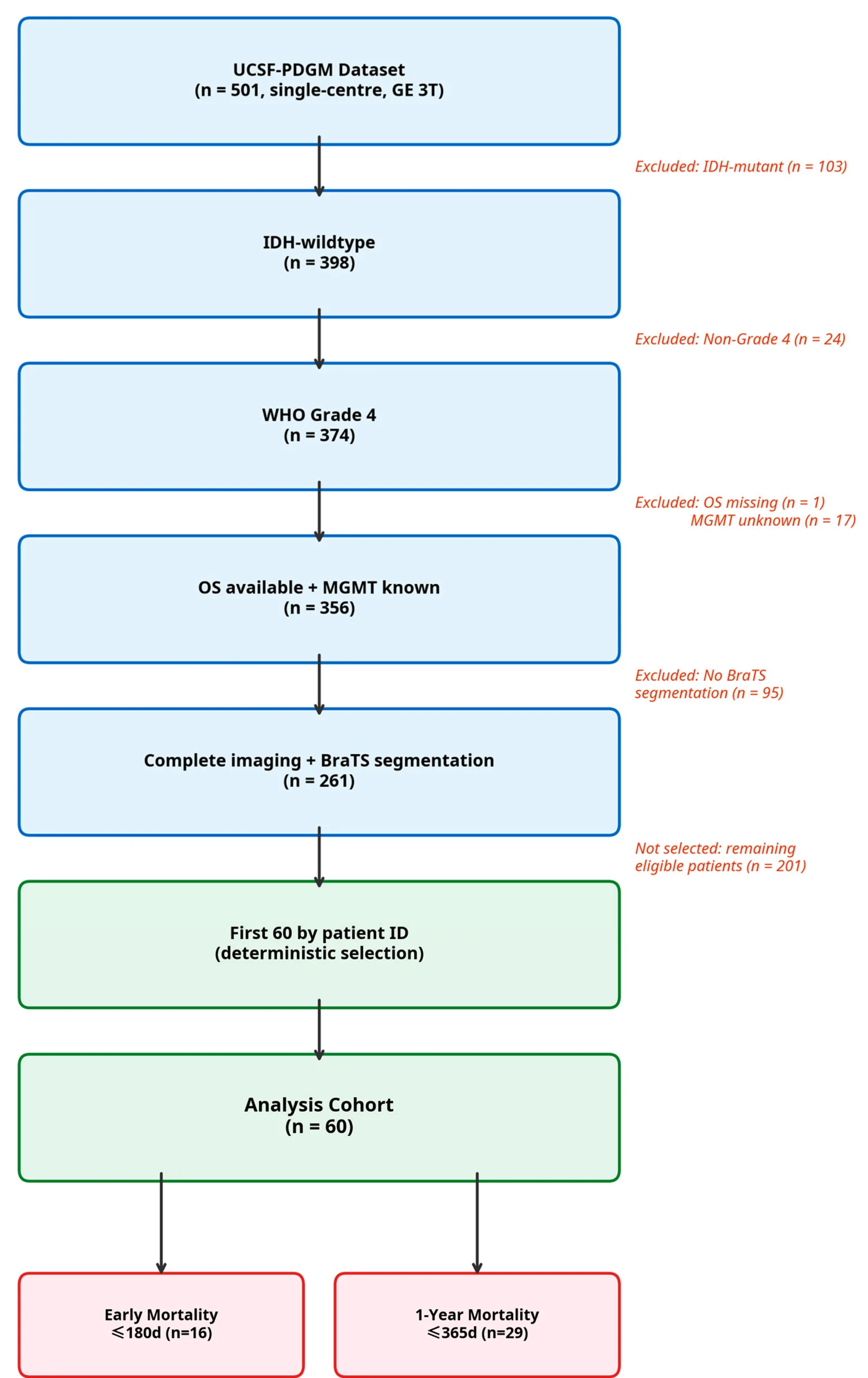
**Patient selection flowchart. Sequential application of inclusion criteria from the UCSF-PDGM dataset (*n* = 501) to the final analysis cohort (*n* = 60). Numbers at each step represent patients remaining after application of the stated criterion.** Study design and nested cross-validation framework. Flowchart illustrating patient selection from the UCSF-PDGM and BraTS 2021 datasets, inclusion/exclusion criteria, feature extraction, preprocessing, and the nested cross-validation structure with outer loop (5-fold × 3 repeats) and inner loop (feature selection + model selection from 3 fixed classifiers: LR, RF, Gradient Boosting).

Cohort assembly (revised Figure 1): From the UCSF-PDGM dataset (*n* = 501 subjects), patients were included if they met all of the following criteria: (1) IDH-wildtype status (*n* = 398, excluding 103 IDH-mutant); (2) WHO CNS Grade 4 (*n* = 374, excluding 24 non-Grade 4); (3) available overall survival data (*n* = 373); (4) known MGMT promoter methylation status (*n* = 356, excluding 17 indeterminate); and (5) complete multimodal MRI with automated tumor segmentation available via the BraTS 2021 challenge (*n* = 261, excluding 95 without segmentation). The final analysis cohort of *n* = 60 was defined as the first 60 eligible patients by ascending dataset identifier. This deterministic selection was performed without reference to outcome values or model performance. In a descriptive comparison with the remaining eligible patients (*n* = 201), age, sex distribution, MGMT methylation, overall survival, and mortality rates were broadly similar, whereas gross total resection was less frequent in the included cohort (43.3% vs. 66.2%; Supplementary Table S7). Because the analysis did not include all eligible records, potential selection bias cannot be excluded.

### 2.2. MRI Acquisition and Preprocessing

All MRI data were acquired using a standardized institutional protocol on a single GE Discovery MR750 3 T scanner. Sequences included T1-weighted (pre-contrast), T1-weighted post-gadolinium (T1ce), T2-weighted, and FLAIR. As part of the BraTS preprocessing pipeline, images were skull-stripped, co-registered to the SRI24 atlas, and resampled to 1 mm isotropic voxels [22]. All radiomic features were extracted from these preprocessed, co-registered images at 1 mm isotropic resolution (matrix 240 × 240 × 155).

### 2.3. Tumor Segmentation

Tumor segmentation masks from BraTS 2021 delineated three subregions: whole tumor (WT; union of all tumor voxels including peritumoral edema/infiltration on FLAIR), tumor core (TC; non-enhancing solid tumor plus enhancing tumor), and enhancing tumor (ET; contrast-enhancing voxels on T1ce). Segmentations were generated by expert neuroradiologists and quality-controlled by the BraTS organizers [22,23].

### 2.4. Radiomic Feature Extraction

Radiomic features were extracted using PyRadiomics v3.0.1 [25] in compliance with the Image Biomarker Standardization Initiative (IBSI) [11]. All seven IBSI-standardized feature classes were enabled. Features were computed from four MRI sequences across three tumor subregions, yielding 1284 initial features per patient (Table 2).

### 2.5. Feature Preprocessing

All preprocessing steps were performed within each training fold to prevent data leakage:

1. Z-score normalization: Mean and standard deviation computed on training data only; applied to both training and test data using training parameters.
2. Variance filter: Features with variance < 0.01 in the training fold were removed.
3. Correlation filter: Among feature pairs with Pearson |r| > 0.95, the feature with lower univariate association with the outcome was removed. This step removes redundant shape duplicates across sequences and highly collinear texture features.

After preprocessing, approximately 120 features remained per fold (exact number varied across folds due to fold-specific filtering).

### 2.6. Nested Cross-Validation Framework

The nested cross-validation structure consisted of two loops (Figure 1):

**Outer loop:** Stratified 5-fold cross-validation, repeated 3 times (15 outer folds total). Stratification ensured proportional representation of early mortality cases in each fold.

Inner loop (within each outer training fold): Feature selection: Mutual information between each feature and the outcome was computed using the outer training data, and the top 25 features were selected. Model selection: The three classifiers with fixed hyperparameters were evaluated using stratified 3-fold cross-validation within the outer training data. The classifier with the highest mean inner-loop AUC was selected, retrained on the complete outer training fold, and then evaluated on the held-out outer test fold.

Classifiers evaluated:—Random Forest (n_estimators = 100; max_depth = 10)—Logistic Regression (C = 1.0; max_iter = 1000)—Gradient Boosting (n_estimators = 100; max_depth = 5)

Performance was evaluated on the held-out outer test fold using the classifier selected during training. Hyperparameters were fixed before the analysis to limit model complexity for the small sample size (*n* = 60, n_events = 16) rather than being optimized on the data. No nested hyperparameter search was performed. All analyses were performed in Python 3.11 using scikit-learn 1.3.0, PyRadiomics 3.0.1, and NumPy 1.24. A single random seed (random_state = 42) was used for the RepeatedStratifiedKFold generator, which deterministically produces all 15 outer fold assignments.

As a post hoc sensitivity analysis, the nested CV pipeline was repeated using shape features alone (42 features) and shape features combined with clinical variables (46 features), using identical outer folds and inner-loop model selection. This analysis assessed whether the predictive signal was dependent on texture features affected by the discretization limitation.

**Comparison condition (standard CV):** Feature selection was performed on the entire dataset (*n* = 60) before outer CV splitting, replicating the methodology of most published radiomics studies. This served as a direct comparison to quantify performance inflation.

### 2.7. Outcome Definitions

**Primary endpoint:** Early mortality, defined as overall survival ≤180 days from the date of diagnosis. Patients were classified as early mortality (*n* = 16) or survivors beyond 180 days (*n* = 44). All patients had either documented death or follow-up ≥180 days.

**Secondary endpoint:** 1-year mortality, defined as death within 365 days from diagnosis. Patients who died within 365 days were classified as events (*n* = 29); patients alive at ≥365 days were classified as non-events (*n* = 31).

### 2.8. Statistical Analysis

Classification performance was assessed using area under the receiver operating characteristic curve (AUC). For each of the 15 outer folds (5-fold × 3 repeats), AUC was computed on the held-out test fold. The primary performance metric is the mean AUC ± standard deviation across 15 outer folds. This fold-level averaging approach provides both a point estimate and a measure of variability across data splits.

The numerical AUC difference between radiomics and clinical-only models was assessed descriptively. Given the small sample size (*n* = 60, 16 events for early mortality) and the dependence structure among repeated CV folds, formal statistical comparison of AUC differences (e.g., DeLong test) was not performed, as such tests require independent predictions and may not hold for cross-validated estimates [26]. Confidence intervals were not computed because bootstrap resampling of fold-level AUCs with only 15 folds would yield unreliable interval estimates. We considered the corrected resampled *t*-test [27] and permutation-based approaches; however, both assume independence of test-set predictions across folds, which is violated in repeated CV on *n* = 60 patients. We therefore report fold-level distributions descriptively rather than applying formal inference with potentially anticonservative type I error control.

The multivariate Cox model was specified with a clinically motivated covariate set: age at diagnosis, extent of resection, and MGMT promoter methylation status were included as established prognostic factors in IDH-wildtype glioblastoma. Whole-tumor surface area was added as the primary radiomic variable of interest based on its high selection frequency across cross-validation folds (13/15 folds). This four-variable model was chosen a priori based on clinical reasoning and feature stability results, rather than through data-driven stepwise selection. Multicollinearity was assessed using variance inflation factors (VIF < 5, considered acceptable; all retained variables had VIF < 3). The proportional hazards assumption was assessed by visual inspection of scaled Schoenfeld residuals. Kaplan–Meier curves were compared using the log-rank test.

Benjamini–Hochberg false discovery rate (FDR) correction was applied to univariate feature-survival associations across all preprocessed features (threshold: q < 0.05) [28]. Cohen’s d was computed comparing early mortality patients (≤180 days, *n* = 16) with long-term survivors (>18 months, *n* = 24).

### 2.9. Software and Reproducibility

We used Python 3.11 with scikit-learn 1.3.0, PyRadiomics 3.0.1, and lifelines 0.29.0. All analyses used publicly available data: BraTS 2021 and UCSF-PDGM v3. This study followed the TRIPOD+AI 2024 reporting guideline for prediction model studies [29]; a completed checklist is provided in Supplementary Table S5. Analysis code is available from the corresponding author upon reasonable request.

## 3. Results

### 3.1. Nested Cross-Validation Reveals Data Leakage

**Figure 2:**
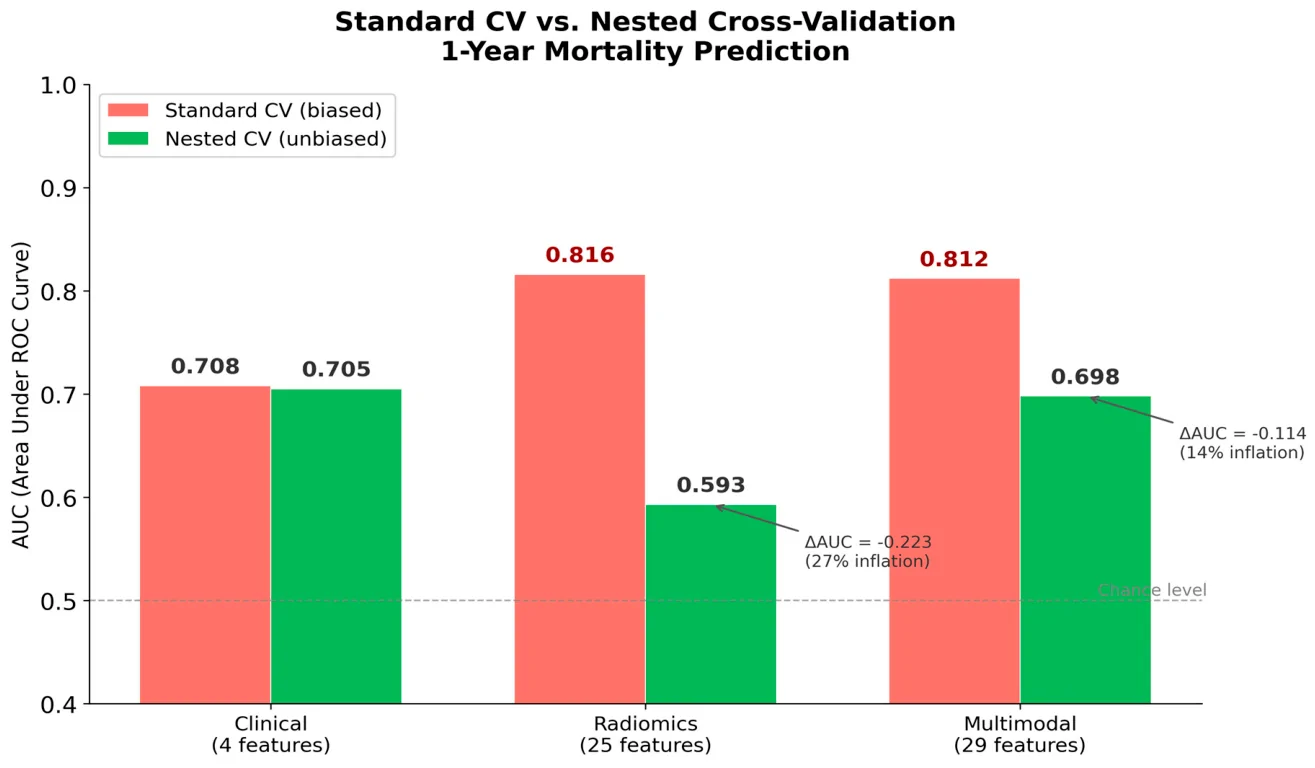
Standard CV versus nested cross-validation performance for 1-year mortality prediction. Standard CV with full-cohort feature selection overestimated radiomics performance (AUC 0.816 → 0.593; ΔAUC = −0.223, 27% inflation). Clinical models remained stable (AUC 0.708 vs. 0.705). Multimodal models showed similar inflation (AUC 0.812 → 0.698; ΔAUC = −0.114, 14% inflation).

Standard cross-validation with feature selection on the full dataset substantially overestimated radiomics performance for 1-year mortality prediction (Table 3, Figure 2).

Across the 15 outer folds, the paired difference in AUC (standard minus leakage-controlled) for early mortality had a median of 0.074 (IQR: 0.018–0.128; range: −0.037 to 0.250), with 12 of 15 folds (80%) showing higher AUC under standard CV. For 1-year mortality, 14 of 15 folds showed higher standard CV performance. These fold-level distributions are reported descriptively; formal inference was not applied due to the correlated fold structure inherent in repeated cross-validation on *n* = 60 patients.

Feature instability analysis revealed substantial variability in feature selection across outer CV folds. Among the top 25 features selected per fold, only WT surface area appeared consistently (≥87% of folds). Most features selected in one-fold were not selected in others, explaining the performance drop under nested CV and highlighting the sensitivity of radiomic feature rankings to data composition.

### 3.2. Early Mortality Prediction

**Figure 3:**
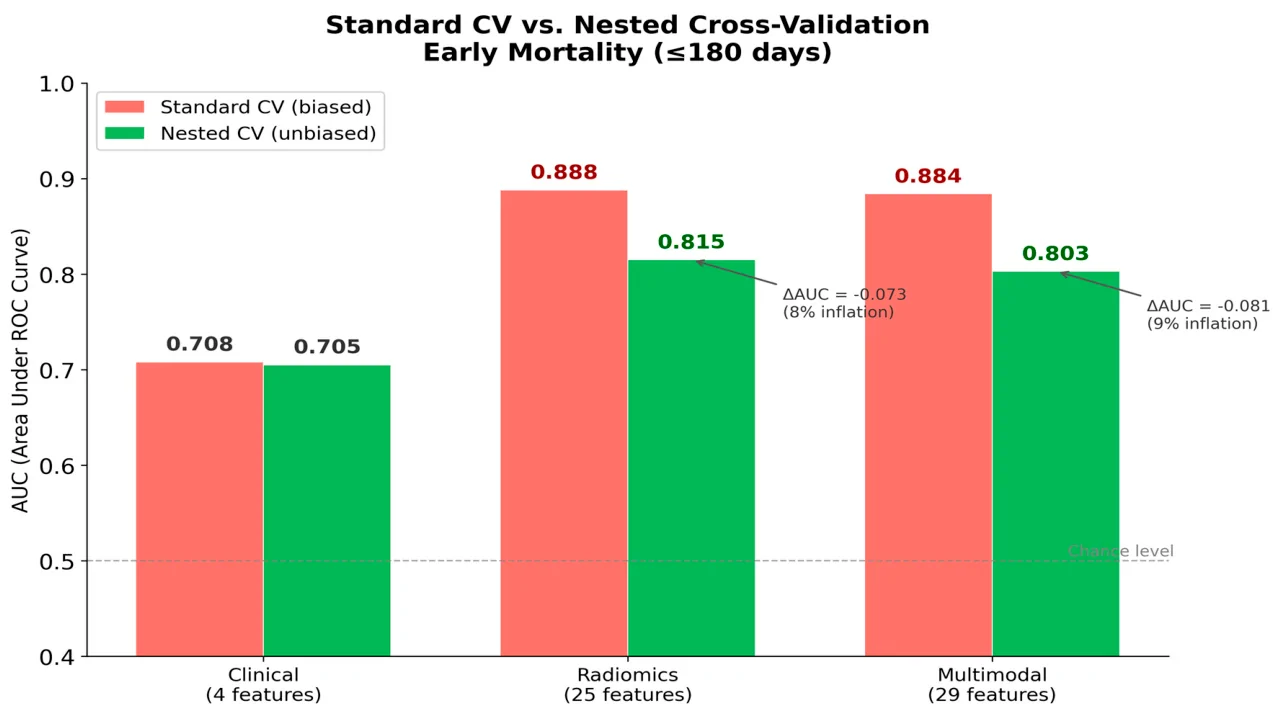
Standard CV versus nested cross-validation performance for early mortality prediction (≤180 days). Same axis scale as in Figure 2 for direct comparison. Standard CV inflated radiomics AUC from 0.815 to 0.888 (8% inflation) and multimodal AUC from 0.803 to 0.884 (9% inflation). Clinical models remained stable (0.708 vs. 0.705).

For the primary endpoint (early mortality, ≤180 days), standard CV similarly inflated radiomics performance compared to nested cross-validation (Table 4, Figure 3).

The pattern of inflation was consistent across both endpoints: clinical models remained stable (no data-driven feature selection), while radiomics and multimodal models showed substantial inflation under standard CV. The magnitude of inflation was smaller for early mortality (8–9%) than for 1-year mortality (14–27%), suggesting that the early mortality endpoint captures a stronger biological signal from baseline imaging that is more robust to data splits.

Under nested CV, radiomics (AUC = 0.815) numerically outperformed the clinical-only model (AUC = 0.705) by ΔAUC = 0.110. Given the small sample size (*n* = 60, 16 events), this difference is imprecisely estimated and does not provide sufficient evidence of superiority over the clinical model; confirmation in larger cohorts is required (Calibration results, including comparison of the observed Brier score with a prevalence-based null model, are reported in Section 3.7).

Radiomics performed substantially better for early mortality (AUC = 0.815) than for 1-year mortality (AUC = 0.593) under nested CV. This endpoint-dependent performance suggests that radiomic features capture aggressive tumor biology that manifests as rapid clinical deterioration, rather than long-term survival, which is confounded by treatment response and non-tumor factors.

### 3.3. Feature Stability Under Cross-Validation

**Figure 4:**
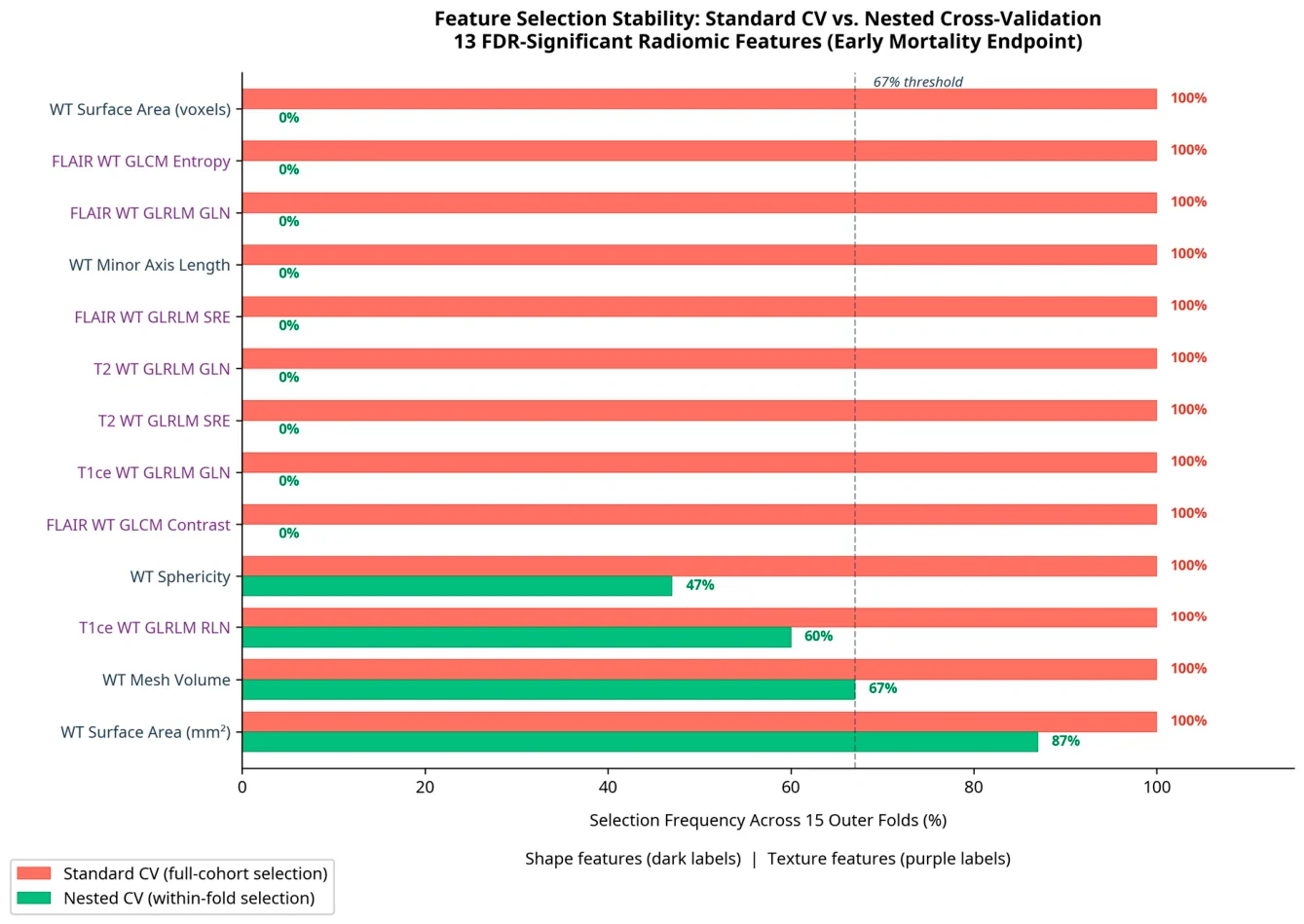
Feature selection stability: standard CV versus nested cross-validation. For each of the 13 FDR-significant features, the selection frequency across 15 outer folds is shown for standard CV (full-cohort feature selection; red bars) and nested CV (within-fold selection; green bars). Under standard CV, all features are selected with perfect consistency (100%) because the same full-cohort feature ranking is applied to every fold—this does not represent an independent empirical stability finding. Under nested CV, selection frequency drops dramatically: WT surface area showed the highest cross-endpoint stability (13/15 folds), followed by mesh volume (10/15 folds); 9 of 13 OS-associated features were never selected for early mortality classification (0%), revealing limited cross-endpoint stability.

To assess the robustness of radiomic features, we compared feature selection stability between standard CV and nested CV across the 15 outer folds (Figure 4).

Among the 13 FDR-significant features, WT surface area in mm^2^ showed the highest cross-endpoint stability (13/15 folds = 87%), followed by WT mesh volume (10/15 folds = 67%). T1ce GLRLM RunLengthNonUniformity appeared in 9/15 folds (60%), and WT sphericity in 7/15 folds (47%). The remaining 9 features—including all FLAIR texture features (GLRLM GLN, GLCM entropy, GLRLM SRE, GLCM contrast), all T2 texture features, T1ce GLRLM GLN, WT surface area in voxels, and WT minor axis length—were never selected in any nested CV fold (0/15 = 0%). Because the 13 features were identified by their association with continuous overall survival, whereas selection frequency was assessed within nested CV folds trained on binary early mortality (≤180 days), this discrepancy reflects limited cross-endpoint stability and substantial dependence on the analytic framework—not solely feature instability per se.

Note regarding surface area features: WT surface area (mm^2^) and WT surface area (voxels) have identical Spearman correlation (ρ = −0.463) and q-value (0.025) with overall survival. These features express the same geometric property in different units. WT surface area (voxels) was removed by the within-fold correlation filter in all 15 folds (Pearson |r| > 0.95 with surface area in mm^2^), explaining its 0% selection frequency despite full-cohort FDR significance.

### 3.4. Exploratory Full-Cohort Feature Association Analysis

The following analysis was performed on the full cohort as an exploratory descriptive assessment of feature-outcome associations. Because the correlation-based redundancy filter uses outcome information to select among correlated features, and the same outcome data are subsequently used for significance testing, the resulting q-values are not strictly inferentially valid. This analysis is presented to characterize the feature space, not to claim validated biomarker status.

After Benjamini–Hochberg FDR correction across all preprocessed features (approximately 120 features surviving preprocessing on the full cohort), 13 radiomic features reached exploratory significance (q < 0.05): 5 shape features and 8 texture features (Table 5).

All correlations with overall survival were negative, indicating that higher feature values were associated with shorter survival. All significant texture features were derived from the whole tumor subregion.

**Important methodological note on texture features:** The PyRadiomics extraction used intensity normalization (normalizeScale = 1), followed by a fixed bin width (binWidth = 25, specifying the intensity width per bin in the discretized image). Because z-score normalization compresses the intensity range to approximately [−3, +3], this combination yields only 1–2 discrete gray levels for most patients. Consequently, gray-level-dependent texture features (GLCM, GLSZM, and GLRLM features involving gray-level variation such as GrayLevelNonUniformity) are reduced to trivial measures in this dataset. FLAIR GLRLM GLN in particular correlates almost perfectly with tumor surface area (Pearson r = 0.999), indicating that it functions as a volume/size surrogate rather than a true texture descriptor under this discretization scheme. Run-length-based features (RunLengthNonUniformity, ShortRunEmphasis) may retain some spatial information because they quantify run-length distributions, but their interpretation also requires caution under coarse discretization. Shape features are unaffected by discretization. Non-histogram first-order measures (mean, median, range) are less affected, whereas histogram-based first-order features (entropy, uniformity) may also depend on the number of discrete bins. This limitation is discussed further in Section 4.7.

### 3.5. Survival Analysis

**Figure 5:**
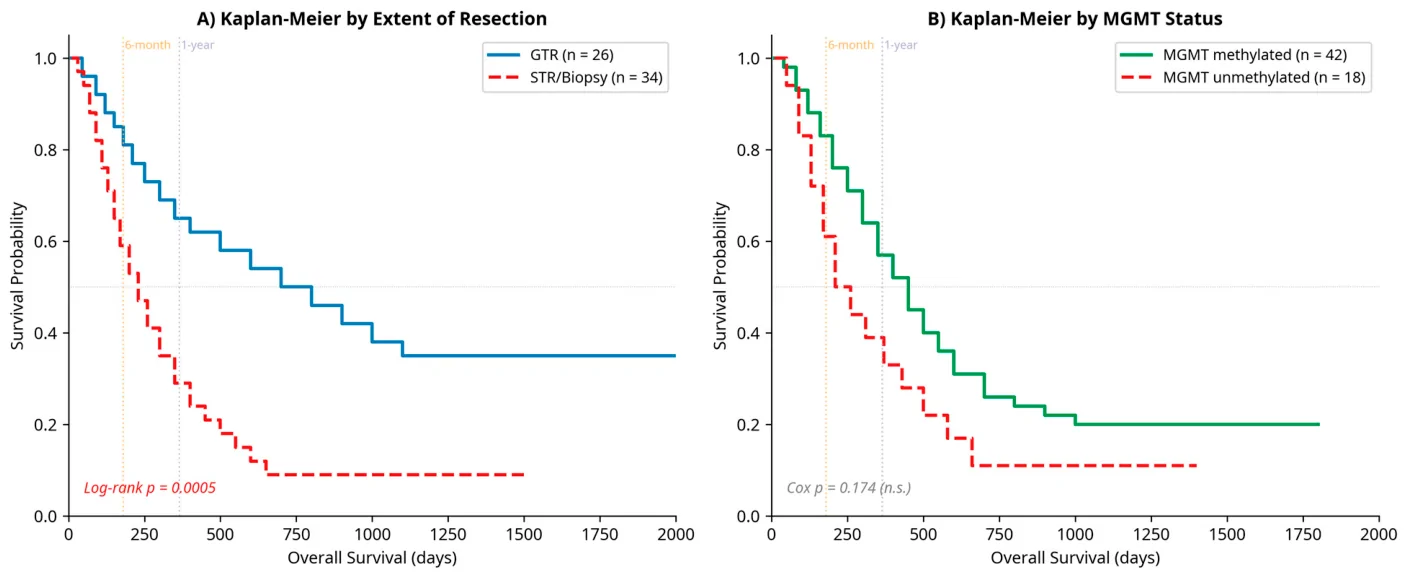
Kaplan–Meier survival curves. (**A**) Stratified by extent of resection: GTR (*n* = 26) versus STR/biopsy (*n* = 34); log-rank *p* = 0.0005. (**B**) Stratified by MGMT promoter methylation status: methylated (*n* = 42) versus unmethylated (*n* = 18); *p* = 0.174 (not significant).

Kaplan–Meier analysis demonstrated that extent of resection was the dominant clinical prognostic factor (Figure 5A). Patients with gross total resection (GTR, *n* = 26) had significantly longer survival than those with subtotal resection or biopsy (STR/biopsy, *n* = 34; log-rank *p* = 0.0005). MGMT promoter methylation status did not reach significance in this IDH-wildtype cohort (*p* = 0.174; Figure 5B). Exploratory univariate and multivariate Cox proportional hazards regression results are summarized in Table 6.

The multivariate Cox model included a pre-specified set of clinically motivated covariates: extent of resection, age, and MGMT promoter methylation status (included a priori as a clinically established prognostic factor). Whole-tumor surface area was included as the primary radiomic variable based on its high selection frequency across cross-validation folds (13/15 folds). FLAIR GLRLM GLN was excluded because post hoc analysis revealed that the discretization parameters (normalizeScale = 1, binWidth = 25) produced only 1–2 gray levels, rendering GLN a near-perfect surrogate of tumor surface area (Pearson r = 0.999). Variance inflation factors for all retained variables were below 3. The proportional hazards assumption was assessed by visual inspection of scaled Schoenfeld residuals; no systematic violations were observed.

Extent of resection was the strongest independent predictor (HR = 0.44, *p* = 0.009), followed by tumor surface area (HR = 1.38, *p* = 0.021) and age (HR = 1.03, *p* = 0.038). MGMT methylation did not reach significance (*p* = 0.654). Whole-tumor surface area remained independently associated with survival after adjustment for clinical variables, suggesting that tumor morphological extent—as quantified by radiomics—captures prognostic information beyond standard clinical assessment.

**Figure 6:**
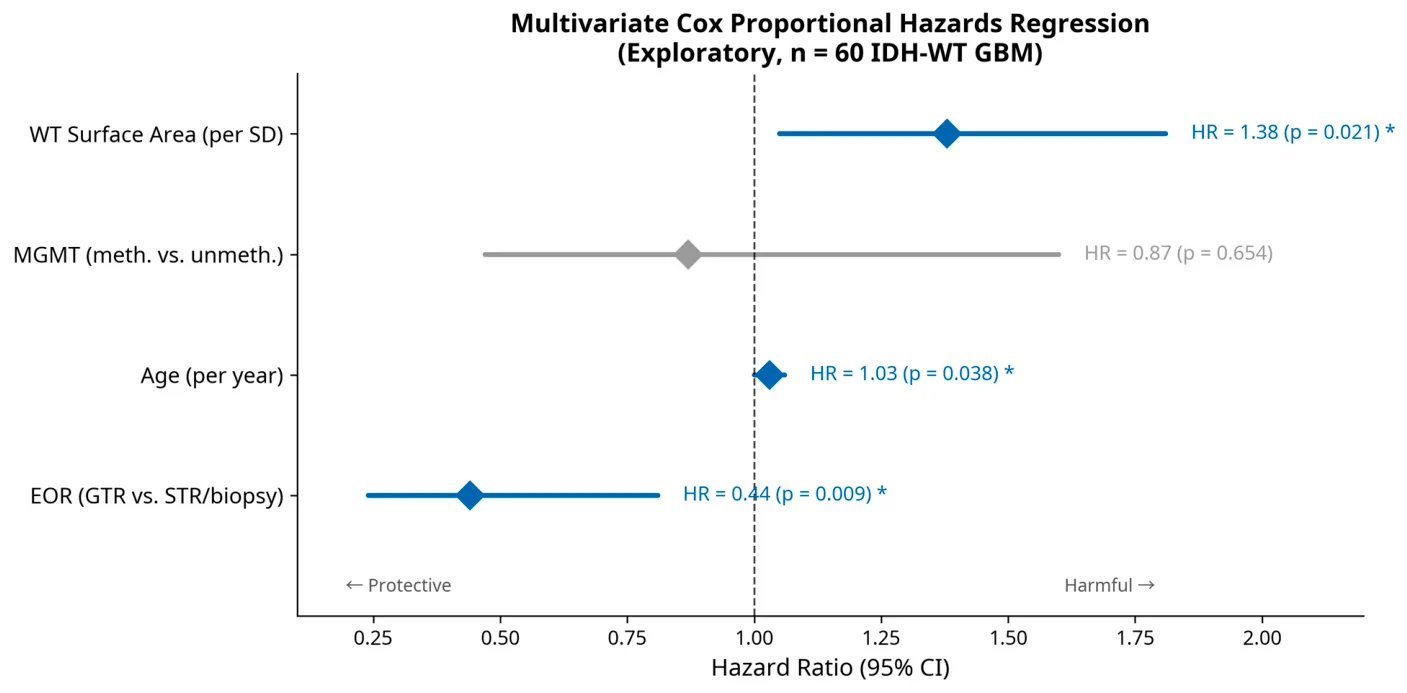
Multivariate Cox proportional hazards regression (exploratory). Forest plot showing hazard ratios with 95% confidence intervals. Extent of resection (GTR vs. STR/biopsy) was the strongest independent predictor (HR = 0.44, *p* = 0.009). Tumor surface area retained significance after adjustment for clinical variables (HR = 1.38, *p* = 0.021). Blue markers and confidence intervals indicate statistically significant associations (*p* < 0.05), whereas grey indicates non-significant associations. Diamonds represent hazard ratio point estimates, horizontal lines indicate 95% confidence intervals, the asterisk denotes *p* < 0.05, and the vertical dashed line marks the null value (HR = 1.0); arrows indicate protective and harmful directions of association.

This Cox analysis is exploratory: Surface area was identified through a full-cohort analysis and its prognostic value requires external validation. The analysis demonstrates that among radiomic features, morphological descriptors (shape features) provide the most robust and interpretable survival associations in this cohort. The adjusted hazard ratios are summarized in Figure 6.

### 3.6. Sensitivity Analysis: Shape-Only Model

To assess whether the predictive signal was driven by morphological features (unaffected by discretization) rather than texture features (potentially uninformative due to coarse gray-level quantization), we repeated the nested CV analysis using only shape features (14 features × 3 subregions = 42 features) and compared against clinical-only and combined models (Table S6).

Shape features alone achieved AUC = 0.873 ± 0.106 for early mortality (using a true inner 3-fold cross-validation loop for classifier selection). Combining shape with clinical features yielded AUC = 0.862 ± 0.099. For 1-year mortality, shape-only achieved 0.763 ± 0.120 and shape + clinical 0.770 ± 0.129, both substantially outperforming the full radiomics model (0.593 ± 0.142). These results confirm that the predictive signal under leakage-controlled CV is predominantly morphological and robust to the discretization limitation.

### 3.7. Limitations of Calibration Assessment

For early mortality prediction, calibration was assessed using the Brier score. The observed Brier score of 0.218 should be interpreted cautiously: given the event prevalence of 26.7% (16/60), a null model assigning the marginal prevalence to all patients would achieve a Brier score of approximately 0.196. The observed Brier score therefore does not demonstrate calibration superiority over a prevalence-based prediction. Formal calibration assessment (calibration slope, calibration-in-the-large) was not performed due to the small sample size. Decision Curve Analysis was not included because net benefit estimates with only 16 events are highly unstable and would not provide reliable clinical utility assessment [30].

## 4. Discussion

### 4.1. Methodological Contribution

This study provides a systematic quantification of performance inflation caused by data leakage in GBM radiomics. Feature selection on the full dataset before cross-validation inflated AUC by up to 0.223 (27%) for radiomics models predicting 1-year mortality, and by 0.073 (8%) for early mortality prediction. Clinical models remained stable because they require no data-driven selection step. This finding empirically confirms theoretical predictions by Varma and Simon [16] and Cawley and Talbot [17] and has direct implications for the interpretation of the published radiomics literature.

The magnitude of inflation (ΔAUC = −0.223 for 1-year mortality; ΔAUC = −0.073 for early mortality) is concerning because many published GBM radiomics studies report AUC values in the range of 0.75–0.85 using standard cross-validation [12,13,14,15]. Our results suggest that a substantial portion of this reported performance may be attributable to data leakage rather than genuine predictive signal. This aligns with recent systematic reviews highlighting poor methodological quality in radiomics studies [18,19].

The smaller inflation for early mortality (8–9%) compared to 1-year mortality (27%) is noteworthy. This difference likely reflects the stronger biological signal captured by radiomic features for acute tumor aggressiveness: features that predict rapid deterioration within 6 months are more directly linked to imaging-visible tumor characteristics than features predicting 1-year survival, which is confounded by treatment response and non-tumor factors.

### 4.2. Feature Stability

Feature stability analysis revealed that among 13 FDR-significant features, WT surface area showed the highest cross-endpoint stability (13/15 folds), followed by mesh volume (10/15 folds), and 9 of 13 were never selected in any fold (0%)—despite perfect selection consistency (100%) under standard CV. This stark contrast illustrates the artificial stability that is mechanically introduced when a fixed full-cohort feature ranking is reused across folds: because the same ranking is applied to all folds, the resulting consistency does not represent an independent empirical finding but rather a direct consequence of the data leakage.

The most stable features under nested CV—WT surface area (13/15 folds) and WT mesh volume (10/15 folds)—are shape features reflecting tumor morphological extent. T1ce GLRLM RunLengthNonUniformity (60%) was the only texture feature with meaningful selection frequency, consistent with its measurement of spatial run-length distributions, which may retain some information despite coarse gray-level discretization. Notably, FLAIR GLRLM GLN was not selected in any nested CV fold (0%) for early mortality classification, despite showing a univariate association with overall survival in the full-cohort analysis. This endpoint-specific discrepancy highlights that feature significance in survival analysis does not guarantee selection stability in classification tasks with different endpoints.

The finding that 9 of 13 FDR-significant features were never selected in any nested CV fold provides a further illustration of the limitations of standard radiomics methodology. Because the FDR analysis targeted continuous OS, while nested CV optimized binary early mortality, this cross-endpoint discrepancy may partly reflect endpoint-specific feature relevance rather than pure instability. Nevertheless, the finding underscores that full-cohort significance should not be equated with selection robustness under rigorous validation. This observation underscores the critical importance of reporting feature stability alongside significance testing in radiomics studies.

### 4.3. Early Mortality Prediction

Despite rigorous nested validation, radiomics achieved AUC = 0.815 for early mortality prediction—a clinically noteworthy result. The endpoint-dependent performance (AUC = 0.815 for early mortality versus 0.593 for 1-year mortality) has a plausible biological explanation. Early mortality may reflect aggressive tumor biology that is captured by baseline imaging-derived features, particularly morphological tumor extent [31]. In contrast, 1-year mortality is confounded by treatment response, surgical complications, comorbidities, and second-line therapies that are not reflected in baseline imaging [32].

The difference between radiomics (AUC = 0.815) and clinical-only models (AUC = 0.705) of ΔAUC = 0.110 is numerically notable and suggests potential added value of radiomics for early mortality prediction. However, given the small sample size and resulting imprecision, this difference requires confirmation in larger, independent cohorts before clinical conclusions can be drawn.

### 4.4. Prognostic Biomarkers and Tumor Size

Tumor surface area emerged as the single most robust radiomic predictor in both univariate analysis (ρ = −0.463, q = 0.025, Cohen’s d = 1.52) and multivariate Cox regression (HR = 1.38, *p* = 0.021 after adjustment for EOR, age, and MGMT). Larger tumors with greater surface area—reflecting irregular, infiltrative morphology—were independently associated with shorter survival. This finding is consistent with the well-established prognostic role of tumor burden in glioblastoma [33,34] and confirms that radiomics can quantify this prognostic factor in a standardized and reproducible manner. The sensitivity analysis (Section 3.6) further corroborates this interpretation: shape features alone achieved AUC = 0.873 for early mortality, exceeding the full radiomics model (AUC = 0.815) by 0.058, while combining shape with clinical features yielded AUC = 0.862. This demonstrates that the predictive signal is predominantly morphological and not dependent on texture features affected by the discretization limitation.

Notably, FLAIR GLRLM gray-Level non-uniformity showed univariate significance (HR = 1.38, *p* = 0.026) but was excluded from the multivariate model because post hoc analysis revealed that the discretization parameters (normalizeScale = 1, binWidth = 25) produced only 1–2 gray levels, rendering GLN a near-perfect surrogate of surface area (r = 0.999). This finding illustrates a critical methodological pitfall: texture features can appear statistically significant while capturing no genuine texture information. Future studies should validate that their discretization parameters produce sufficient gray levels (≥16) before interpreting texture-based associations biologically [35,36,37].

### 4.5. MGMT Methylation in IDH-Wildtype Cohorts

MGMT promoter methylation status did not reach significance in our cohort (univariate *p* = 0.174; multivariate *p* = 0.654). This finding is consistent with emerging evidence that MGMT prognostic value may be attenuated in strictly IDH-wildtype populations [38]. In mixed glioma cohorts, MGMT significance is partly driven by the correlation between IDH mutation and MGMT methylation. In a pure IDH-wildtype cohort, this confounding is eliminated, potentially reducing the apparent prognostic effect. Alternatively, our sample size (*n* = 60) may be insufficient to detect the MGMT effect. With *n* = 60 and 42 MGMT-methylated patients (70%), a two-sided log-rank test at α = 0.05 would have approximately 45% power to detect a hazard ratio of 0.60, assuming median survival of 15 months in the unmethylated group. The non-significant MGMT effect is therefore consistent with both a true null effect and insufficient statistical power.

### 4.6. Comparison with the Published Literature

Table 7 provides narrative context rather than direct benchmarking: endpoint definitions, event rates, sample sizes, and validation methodologies differ across studies. Our leakage-controlled AUC was 0.815 for early mortality (≤6 months) and 0.593 for 12-month mortality. These values should therefore not be interpreted as directly comparable to prior studies reporting 12-month overall-survival performance under different validation strategies.

Recent studies further reinforce both the promise of MRI-based prognostication and the need for rigorous validation. A contemporary review of MRI radiomics in glioblastoma emphasizes persistent challenges related to standardization, reproducibility, and clinical translation [40]. In IDH-wildtype glioblastoma, multiregional multiparametric MRI radiomics has shown prognostic potential [41], while clinical-radiomics survival models have also been evaluated in an independent validation cohort [42]. Complementarily, the multicenter GRASP study demonstrated that MRI-based AI survival prediction can be evaluated across heterogeneous institutions using prospective external test data [43]. Collectively, these studies support continued development of imaging biomarkers while underscoring that reported performance is inseparable from the validation design used to estimate it.

### 4.7. Limitations

This study has several limitations.

First, the sample size (*n* = 60, 16 early mortality events) limits statistical power and results in imprecise performance estimates. With approximately 3–4 events per outer test fold, individual fold AUC values are highly variable. The reported mean AUC should be interpreted as a point estimate with substantial uncertainty. Larger cohorts (*n* > 200) are needed to confirm these findings with adequate precision [29].

Second, all imaging was acquired on a single GE 3 T scanner. Generalizability to other vendors (Siemens, Philips) and field strengths (1.5 T) is unknown; imaging protocol harmonization may be required [44].

Third, nested cross-validation provides leakage-controlled internal estimates but does not substitute for independent external validation on a geographically and temporally distinct cohort [45].

Fourth, treatment details beyond EOR (radiation dose, temozolomide cycles, second-line therapies) were not available. The endpoint “early mortality” therefore reflects death from any cause within 180 days and cannot be equated with treatment failure, as some patients may not have received full standard-of-care therapy.

Fifth, the mutual information-based feature selection method may not be optimal; comparison with LASSO or recursive feature elimination is warranted [46].

Sixth, segmentation masks from BraTS 2021 were used without quantification of inter-observer variability [47].

Seventh, several FDR-significant features—including whole-tumor surface area, mesh volume, and minor axis length—are fundamentally related to tumor size and spatial extent. Tumor burden is a well-established prognostic factor in glioblastoma [33,34]. In our cohort, tumor volumes ranged widely (whole tumor: 3.7–225.5 cm^3^, median 68.1 cm^3^). In the multivariate Cox model (excluding FLAIR GLN due to near-perfect collinearity with surface area, r = 0.999), whole-tumor surface area remained independently associated with survival (HR = 1.38, *p* = 0.021) after adjustment for age, extent of resection, and MGMT status. Future studies with larger cohorts and appropriate texture discretization should formally compare size-only models against texture-based radiomics to disentangle the independent contributions of tumor burden and imaging heterogeneity.

Eighth, the classifier selection within each fold was based on training-fold cross-validated performance. While this prevents direct test-fold leakage, the limited hyperparameter search (three fixed classifiers) may not represent optimal model development. More comprehensive nested designs with full grid search in the inner loop should be explored in future work. Although SHAP provides a widely used framework for feature-level attribution of model predictions [48], a reproducible fold-wise aggregation strategy was not pre-specified in the present study; therefore, SHAP-based interpretability results were not retained in the final analysis.

Ninth, the intensity normalization and discretization parameters (normalizeScale = 1, binWidth = 25) resulted in only 1–2 discrete gray levels for most patients across all MRI sequences. This renders gray-level-dependent texture features (GLCM, GLSZM, and gray-level-based GLRLM features) essentially uninformative as true texture descriptors. FLAIR GLRLM GrayLevelNonUniformity, which showed univariate Cox significance, correlates, r = 0.999, with tumor surface area and functions as a volume surrogate rather than a texture measure. Run-length-based features (RunLengthNonUniformity, ShortRunEmphasis) may retain some spatial information despite coarse gray-level discretization, as they quantify run-length distributions rather than gray-level variation directly; however, their interpretation also requires caution. Shape features are unaffected; non-histogram first-order measures (mean, range) are less affected, whereas histogram-based first-order features (entropy, uniformity) may also depend on discretization. This discretization error does not invalidate the primary finding (leakage quantification) or the shape-based predictive signal, but it means that the texture-specific biological interpretations in this study should be viewed with caution. Future radiomics studies should carefully validate that their discretization parameters produce a sufficient number of gray levels (≥16) to ensure meaningful texture computation [37]. The observed inflation magnitudes represent descriptive estimates specific to this dataset and pipeline configuration. Because the full 1284-feature set included texture features rendered largely uninformative by the discretization artifact, the standard CV pipeline may have preferentially selected from noise features—a scenario in which data leakage is particularly effective at inflating apparent performance. The magnitude of inflation should therefore not be generalized as a universal estimate for radiomics pipelines. Re-extraction with corrected discretization parameters was considered but not performed because: (1) the primary objective was to quantify the methodological effect of feature-selection leakage under controlled conditions using identical feature spaces; (2) introducing corrected discretization would constitute a new analytical pipeline; (3) the shape-only sensitivity analysis provides a parameter-free robustness check (AUC = 0.873). A corrected texture analysis represents a necessary validation step for future work.

More broadly, MRI radiomics is sensitive to preprocessing choices beyond the specific configuration used here. Quantitative MRI work has shown that discretization strategy materially influences radiomic feature reproducibility [49], and a systematic review identified voxel resampling, normalization, and discretization as major determinants of feature robustness [50]. In brain MRI, scanner-related effects may persist despite image-level preprocessing, while harmonization approaches can improve reproducibility across acquisition settings [51]. These observations further support prospective specification and transparent reporting of preprocessing, discretization, and harmonization parameters in future validation studies.

### 4.8. Clinical Implications

Researchers and reviewers should consider requiring nested cross-validation or an equivalent leakage-controlled validation strategy for radiomics studies in which feature selection or model selection is data-driven. If the early mortality prediction observed here (AUC = 0.815) is confirmed in independent multicenter cohorts, potential applications may include risk stratification for clinical-trial enrichment and individualized patient counseling. Before clinical deployment, however, models require prospective evaluation of calibration, incremental value beyond established clinical and molecular predictors, and robustness across scanners, institutions, and acquisition protocols. No direct treatment decisions should be based on the present exploratory findings.

More broadly, imaging-derived prognostic biomarkers should be viewed as one component of precision neuro-oncology rather than as stand-alone decision tools. Emerging therapeutic approaches, including nanomedicine-based drug-delivery strategies intended to address barriers to effective treatment of primary and secondary brain tumors, illustrate the expanding translational landscape in which future imaging biomarkers may operate [52]. Radiomics could potentially contribute to such frameworks through non-invasive phenotyping and risk stratification, but the present study does not establish treatment-selection utility.

## 5. Conclusions

Standard radiomics pipelines can overestimate predictive performance when data-driven feature selection is performed before cross-validation. In this dataset, the descriptive AUC difference between standard and leakage-controlled validation reached 0.223 for 1-year mortality and 0.073 for early mortality. Whole-tumor surface area showed the highest cross-endpoint selection stability (13/15 folds), followed by mesh volume (10/15 folds), whereas 9 of 13 exploratory OS-associated features were never selected for early mortality classification. Radiomics achieved an AUC of 0.815 for early mortality, compared with 0.705 for the clinical-only model. Tumor surface area remained independently associated with survival in exploratory Cox regression (HR = 1.38, *p* = 0.021). Importantly, the shape-only sensitivity analysis achieved AUCs of 0.873 for early mortality and 0.763 for 1-year mortality, indicating that the most reproducible predictive signal in this analysis was predominantly morphological rather than dependent on texture features affected by coarse discretization.

The principal implication is therefore methodological rather than a claim of a clinically ready prognostic classifier. Future studies should keep all data-driven preprocessing, feature-selection, and model-selection steps within the resampling structure; report feature-selection stability; and benchmark high-dimensional radiomics against simpler morphology-based models. Before clinical use, the observed early mortality discrimination must be confirmed in larger, independent multicenter cohorts with prospectively specified preprocessing, appropriate gray-level discretization, scanner harmonization, and formal calibration assessment [48,49,50,51]. External validation should also determine whether radiomics provides clinically meaningful incremental information beyond age, extent of resection, MGMT status, and other established predictors. If validated, imaging biomarkers may contribute to risk stratification and trial enrichment within a broader precision neuro-oncology framework, potentially complementing evolving therapeutic and drug-delivery strategies rather than determining treatment in isolation [52]. Thus, the present findings support leakage-controlled radiomics as a hypothesis-generating and risk-stratification approach that warrants further validation, not as a basis for individual treatment decisions.

## Figures and Tables

**Table 1:** Patient characteristics.

Characteristic	Value
Patients, *n*	60
Sex, male/female	40 (66.7%)/20 (33.3%)
Age, years, mean ± SD (range)	59.3 ± 12.4 (33–84)
Extent of resection: GTR/STR/biopsy	26 (43.3%)/31 (51.7%)/3 (5.0%)
MGMT promoter methylated	42 (70.0%)
Median overall survival, days (range)	398 (14–1907)
Deceased at last follow-up	40 (66.7%)
Early mortality (≤180 days)	16 (26.7%)
1-year mortality (≤365 days)	29 (48.3%)
**Tumor volumes**	
Whole tumor, cm^3^, median (IQR)	68.1 (25.0–118.5)
Tumor core, cm^3^, median (IQR)	21.6 (9.5–27.1)
Enhancing tumor, cm^3^, median (IQR)	15.4 (7.4–20.6)
Maximum 3D diameter (TC), mm, median (IQR)	42.3 (28.7–55.1)

**Table 2:** Radiomic feature classes and extraction parameters.

Feature Class	Features per Combination	Sequence-Dependent	Subregions	Total Columns
Shape	14	No *	3	168 †
First-order	18	Yes	4 × 3	216
GLCM	24	Yes	4 × 3	288
GLRLM	16	Yes	4 × 3	192
GLSZM	16	Yes	4 × 3	192
GLDM	14	Yes	4 × 3	168
NGTDM	5	Yes	4 × 3	60
**Total**	**107**			**1284**

* Shape features are computed from the binary segmentation mask and are therefore sequence-independent (identical values across sequences for the same subregion). † Shape features were extracted for each sequence–subregion combination for pipeline consistency; redundant duplicates were removed during preprocessing by the correlation filter. PyRadiomics extraction parameters: bin width = 25 intensity units per bin; resampled pixel spacing = 1 × 1 × 1 mm (isotropic); interpolator = B-spline; intensity normalization enabled (scale = 1); 3D extraction (force2D = False). The complete PyRadiomics configuration is provided in Supplementary Table S4.

**Table 3:** AUC comparison: standard CV versus nested cross-validation for 1-year mortality.

Model	Standard CV (AUC ± SD)	Nested CV (AUC ± SD)	ΔAUC	Inflation
Clinical (4 features)	0.708 ± 0.171	0.705 ± 0.173	−0.003	0%
Radiomics (25 features)	0.816 ± 0.118	0.593 ± 0.142	−0.223	27%
Multimodal (29 features)	0.812 ± 0.121	0.698 ± 0.134	−0.114	14%

Clinical features: age, sex, MGMT methylation status, extent of resection. Clinical models showed negligible performance change (AUC 0.708 vs. 0.705) because clinical features require no data-driven selection. Radiomics and multimodal models showed substantial inflation because feature selection on the full dataset allowed test-set information to influence the selection step.

**Table 4:** Standard CV versus nested CV performance for early mortality prediction.

Model	Standard CV (AUC ± SD)	Nested CV (AUC ± SD)	ΔAUC	Inflation
Clinical-only (4 features)	0.708 ± 0.171	0.705 ± 0.173	−0.003	0%
Radiomics	0.888 ± 0.107	0.815 ± 0.087	−0.073	8%
Multimodal	0.884 ± 0.101	0.803 ± 0.109	−0.081	9%

**Table 5:** FDR-significant radiomic features.

**Feature**	**Category**	**Sequence**	**Subregion**	**ρ with OS**	**q-Value**
Surface area (mm^2^)	Shape	—	WT	−0.463	0.025
Surface area (voxels)	Shape	—	WT	−0.463	0.025
Mesh volume	Shape	—	WT	−0.456	0.025
Sphericity	Shape	—	WT	−0.420	0.025
Minor axis length	Shape	—	WT	−0.413	0.025
GLRLM GLN	Texture	FLAIR	WT	−0.425	0.040
GLCM entropy	Texture	FLAIR	WT	−0.418	0.040
GLRLM SRE	Texture	FLAIR	WT	−0.405	0.040
GLRLM RLN	Texture	T1ce	WT	−0.399	0.040
GLRLM GLN	Texture	T1ce	WT	−0.392	0.040
GLRLM SRE	Texture	T2	WT	−0.391	0.040
GLRLM GLN	Texture	T2	WT	−0.390	0.040
GLCM contrast	Texture	FLAIR	WT	−0.389	0.040

**Table 6:** Exploratory Cox proportional hazards regression.

Variable	Univariate HR (95% CI)	*p*	Multivariate HR (95% CI)	*p*
EOR (GTR vs. STR/biopsy)	0.63 (0.47–0.86)	0.004	0.44 (0.24–0.81)	0.009
Age (per year)	1.02 (1.00–1.04)	0.031	1.03 (1.00–1.06)	0.038
MGMT (methylated vs. unmeth.)	0.78 (0.43–1.43)	0.174	0.87 (0.47–1.60)	0.654
WT surface area (per SD)	1.44 (1.10–1.88)	0.007	1.38 (1.05–1.81)	0.021

**Table 7:** Comparison with published GBM radiomics studies.

Study	Year	*n*	Validation	AUC	Endpoint	Nested CV	Public Data
Kickingereder et al. [12]	2016	119	LOOCV	0.79	12-mo OS	No	No
Lao et al. [13]	2017	75	10-fold CV	0.74	12-mo OS	No	No
Choi et al. [14]	2020	126	External	0.85	12-mo OS	No	No
Karabacak et al. [15]	2024	865	5-fold CV	0.80	OS (cont.)	No	Yes
Umehara et al. [39]	2026	278	External	—	OS	No	No
This study	2026	60	Nested CV	0.815	≤6-mo mortality	Yes	Yes
This study	2026	60	Nested CV	0.593	12-mo mortality	Yes	Yes

## Data Availability

The datasets analyzed in this study are publicly available: BraTS 2021 and UCSF-PDGM v3 (https://www.kaggle.com/datasets/usmansadiqcs/ucsf-pdgm-v3-dataset; 8 August 2026). Analysis code is available from the corresponding author upon reasonable request.

## Supplementary Materials

The following supporting information can be downloaded at: https://www.mdpi.com/article/10.3390/cancers18162595/s1, Table S1: Nested Cross-Validation Pseudocode; Table S2: Classifier Specifications and Inner-Loop Model Selection; Table S3: Early Mortality Group Characteristics; Table S4: Complete PyRadiomics Configuration; Table S5: TRIPOD+AI 2024 Reporting Checklist; Table S6: Sensitivity Analysis—Shape-Only Nested Cross-Validation Results; Figure S1: Tumor Morphology and Survival Scatter Plots; Figure S2: Distribution of AUC Values Across 15 Outer Folds; Table S7: Comparison of Included (*n* = 60) and Excluded (*n* = 201) Eligible Patients.

## Abbreviations

**Abbreviation**	**Meaning**
AI	artificial intelligence
AUC	area under the receiver operating characteristic curve
CI	confidence interval
CNS	central nervous system
CV	cross-validation
EOR	extent of resection
ET	enhancing tumor
FDR	false discovery rate
FLAIR	fluid-attenuated inversion recovery
GBM	glioblastoma
GLDM	gray level dependence matrix
GLCM	gray level co-occurrence matrix
GLN	gray level non-uniformity
GLRLM	gray level run length matrix
GLSZM	gray level size zone matrix
GTR	gross total resection
HR	hazard ratio
IBSI	Image Biomarker Standardization Initiative
IDH	isocitrate dehydrogenase
IQR	interquartile range
LR	logistic regression
MGMT	O6-methylguanine-DNA methyltransferase
MRI	magnetic resonance imaging
NGTDM	neighboring gray tone difference matrix
OS	overall survival
RF	random forest
RLN	run length non-uniformity
SD	standard deviation
SHAP	SHapley Additive exPlanations
SRE	short run emphasis
STR	subtotal resection
T1ce	contrast-enhanced T1-weighted imaging
TC	tumor core
TRIPOD+AI	Transparent Reporting of a multivariable prediction model for Individual Prognosis Or Diagnosis + Artificial Intelligence
UCSF-PDGM	University of California San Francisco Preoperative Diffuse Glioma MRI dataset
VIF	variance inflation factor
WHO	World Health Organization
WT	whole tumor
