# Supplementary material for: Nested Cross-Validation Reveals Performance Inflation in MRI Radiomics for Early Mortality Prediction in IDH-Wildtype Glioblastoma
Source: Cancers (Basel). 2026 Aug 12;18(16):2595. doi: 10.3390/cancers18162595 (PMC13510728; doi:10.3390/cancers18162595)
Supplement: Supplementary file 1 [file cancers-18-02595-s001.zip › cancers-4473733-supplementary.pdf]

Supplementary Materials

Nested Cross-Validation Reveals Performance Inflation in MRI Radiomics for Early Mortality Prediction in IDH-Wildtype Glioblastoma

Lucas I. Becker[1]*, Nicolas Noel Neidert[2], Roberto Doria-Medina[2], Manou Overstijns[2], Maryam Wendeberg[1], Urs Würtemberger[3], Horst Urbach[1]

[1] Department of Neuroradiology, University Medical Centre Freiburg, Freiburg, Germany

[2] Department of Neurosurgery, University Medical Centre Freiburg, Freiburg, Germany

[3] Raya Diagnostics, Munich, Germany

** Corresponding author: Lucas I. Becker; lucas.becker@uniklinik-freiburg.de*

Contents: Tables S1-S7 and Figures S1-S2

All numerical values, methodological specifications, and interpretations are aligned with the submitted manuscript.

Table S1. Nested Cross-Validation Pseudocode

```
Algorithm: Repeated nested cross-validation with within-fold feature and model selection

Input: feature matrix X (n = 60, p = 1284), outcome y, outer folds = 5, repeats = 3,
       inner folds = 3

For each repeat r = 1,...,3:
    Create a stratified 5-fold outer split.
    For each outer fold k = 1,...,5:
        Define outer training data (4/5) and held-out outer test data (1/5).

        PREPROCESSING - fit on outer training data only:
        1. Z-score normalization:
           estimate mean and SD on training data and transform both training and test data
           with the training-fold parameters.
        2. Variance filter:
           remove features with training-fold variance < 0.01.
        3. Correlation filter:
           for feature pairs with Pearson |r| > 0.95, remove the feature with the
           lower univariate association with the outcome.

        FEATURE SELECTION - outer training data only:
        4. Compute mutual information between each retained feature and y_train.
        5. Select the 25 features with the highest mutual information.

        INNER MODEL SELECTION:
        6. Evaluate Logistic Regression, Random Forest, and Gradient Boosting
           by stratified 3-fold CV within the outer training data.
        7. Select the classifier with the highest mean inner-loop AUC.

        OUTER EVALUATION:
        8. Retrain the selected classifier on the complete outer training fold.
        9. Predict probabilities for the held-out outer test fold and calculate AUC.
       10. Record the outer-fold AUC and selected features.

Output: mean AUC ± SD across 15 outer folds (5 folds × 3 repeats).

Standard-CV comparison:
    Feature selection is performed once on the full cohort before outer CV splitting.
    The same full-cohort feature ranking is reused across folds, intentionally reproducing
    the leakage-prone condition used to quantify performance inflation.
```

Sensitivity analyses used the same nested-CV structure with shape-only features (42 features) and shape plus clinical variables (46 features).

Table S2. Classifier Specifications and Inner-Loop Model Selection

Classifier	scikit-learn class	Fixed parameters stated in the manuscript
**Logistic Regression**	Logistic Regression	C = 1.0; max_iter = 1000
**Random Forest**	Random Forest Classifier	n_estimators = 100; max_depth = 10
**Gradient Boosting**	GradientBoostingClassifier	n_estimators = 100; max_depth = 5

Within each outer training fold, all three classifiers were evaluated using stratified 3-fold cross-validation. The classifier with the highest mean inner-loop AUC was selected, retrained on the full outer training fold, and evaluated on the held-out outer test fold. The analysis used three fixed classifier specifications rather than an exhaustive hyperparameter search.

Table S3. Early Mortality Group Characteristics

Characteristic	Value reported in the submitted manuscript
**Early mortality group**	16/60 patients (26.7%); overall survival ≤180 days from diagnosis
**Survivors beyond 180 days**	44/60 patients (73.3%); survival beyond 180 days
**Outcome ascertainment**	All patients had documented death or follow-up ≥180 days
**Age, years, mean ± SD (range)**	59.3 ± 12.4 (33-84)
**Sex, male / female**	40 (66.7%) / 20 (33.3%)
**Extent of resection: GTR / STR / biopsy**	26 (43.3%) / 31 (51.7%) / 3 (5.0%)

Characteristic	Value reported in the submitted manuscript
**MGMT promoter methylated**	42 (70.0%)
**Median overall survival, days (range)**	398 (14-1907)
**1-year mortality (≤365 days)**	29/60 patients (48.3%)

Note: Endpoint-stratified sample sizes and the overall cohort characteristics are reproduced exactly as stated in the submitted manuscript. The manuscript does not separately report the remaining clinical covariates by 180-day outcome group.

Table S4. Complete PyRadiomics Configuration

Parameter	Value	Description
**Software**	PyRadiomics v3.0.1	IBSI-compliant radiomic feature extraction
**Input image preprocessing**	BraTS 2021 pipeline	Skull stripping, co-registration to the SRI24 atlas, and 1-mm isotropic resampling
**Image matrix**	240 × 240 × 155	Preprocessed, co-registered image matrix
**MRI sequences**	T1w, T1ce, T2w, FLAIR	Four preoperative MRI sequences
**Tumor subregions**	WT, TC, ET	Whole tumor, tumor core, and enhancing tumor
**binWidth**	25 intensity units	Fixed bin width used for discretization
**resampledPixelSpacing**	[1, 1, 1] mm	Isotropic spatial resolution
**interpolator**	B-spline	Interpolation used for resampling
**normalize**	True	Intensity normalization enabled
**normalizeScale**	1	Normalization scale
**force2D**	False	Full 3D feature extraction
**Feature classes**	All 7 IBSI classes	Shape, first-order, GLCM, GLRLM, GLSZM, GLDM, and NGTDM

Feature classes and extraction combinations

Feature class	Features per sequence-subregion combination	Sequence-dependent	Technical columns
**Shape**	14	No*	168†
**First-order**	18	Yes	216
**GLCM**	24	Yes	288
**GLRLM**	16	Yes	192
**GLSZM**	16	Yes	192
**GLDM**	14	Yes	168
**NGTDM**	5	Yes	60
**Total**	107	-	1284

* Shape features are calculated from the binary segmentation mask and are sequence-independent for a given subregion. † For pipeline consistency, shape features were technically extracted for each sequence-subregion combination; redundant duplicates were removed by the correlation filter.

Subregion definitions: WT = labels 1 + 2 + 4 (peritumoral edema/infiltration, necrotic/non-enhancing core, and enhancing tumor); TC = labels 1 + 4; ET = label 4.

Methodological note on discretization: With normalizeScale = 1 and binWidth = 25, most regions contained only 1-2 discrete gray levels. Gray-level-dependent texture features therefore require cautious interpretation; shape features are unaffected.

Table S5. TRIPOD+AI 2024 Reporting Checklist

Reporting item	Manuscript location	Status / details
Title identifies a prediction-model study	Title; Simple Summary; Abstract	Reported
Background and study objectives	Sections 1 and 4.1	Reported
Source of data, setting, and study dates	Section 2.1	UCSF-PDGM v3; single center; 2015-2021
Eligibility and exclusion criteria	Section 2.1	Reported
MRI acquisition and preprocessing	Section 2.2	Reported
Tumor segmentation	Section 2.3	BraTS 2021 WT, TC, and ET masks
Predictor definition and radiomic extraction	Sections 2.4-2.5; Table 2; Table S4	Reported
Outcome definition and timing	Section 2.7	Early mortality ≤180 days; 1-year mortality ≤365 days
Sample size and event counts	Section 2.1; Table 1; Section 4.7	n = 60; 16 early events; 29 one-year events
Missing data and outcome ascertainment	Section 2.1	Complete eligible cases; all patients had adequate endpoint ascertainment
Model development procedure	Section 2.6; Table S1	Repeated nested CV with within-fold preprocessing and feature selection
Inner-loop model selection	Section 2.6; Table S2	Stratified 3-fold CV among three fixed classifiers
Validation strategy	Section 2.6	5-fold outer CV repeated three times
Performance measures	Section 2.8	AUC and Brier score
Calibration assessment	Section 3.7	Brier score reported; limitations explicitly discussed
Model interpretability	Revised manuscript	Exploratory SHAP analysis removed during revision because a reproducible fold-wise aggregation procedure was not retained.
Participant and cohort results	Table 1; Sections 3.1-3.7	Reported
Model performance results	Tables 3-4 and S6; Figures 2-3 and S2	Mean AUC ± SD across 15 outer folds
Interpretation and clinical implications	Sections 4.1-4.8	Reported with cautious clinical framing
Limitations and generalizability	Sections 3.7 and 4.7	Reported
Data and code availability	Section 2.9; Data Availability Statement	Public datasets; code on reasonable request
Funding and conflicts of interest	End matter	Reported

Note: Study-specific reporting summary. This is a development-only study with internal validation; items that apply exclusively to independent external validation are not applicable.

Table S6. Sensitivity Analysis - Shape-Only Nested Cross-Validation Results

A. Early mortality (≤180 days)

Feature set	N features	AUC ± SD
**Clinical-only**	4	0.705 ± 0.173
**Shape-only**	42	0.873 ± 0.106
**Shape + clinical**	46	0.862 ± 0.099
**Full radiomics**	1284	0.815 ± 0.087
**Full multimodal model**	29	0.803 ± 0.109

B. 1-year mortality (≤365 days)

Feature set	N features	AUC ± SD
**Clinical-only**	4	0.705 ± 0.173
**Shape-only**	42	0.763 ± 0.120
**Full radiomics**	1284	0.593 ± 0.142
**Full multimodal model**	29	0.698 ± 0.134
**Shape + clinical**	46	0.770 ± 0.129

Shape-only features achieved AUC = 0.873 for early mortality, exceeding the full radiomics model (0.815), while shape plus clinical features achieved AUC = 0.862. For 1-year mortality, shape-only (0.763) and shape plus clinical (0.770) both outperformed the full radiomics model (0.593), supporting a predominantly morphological predictive signal that is independent of the intensity-discretization limitation.

Figure S1. Tumor Morphology and Survival Scatter Plots

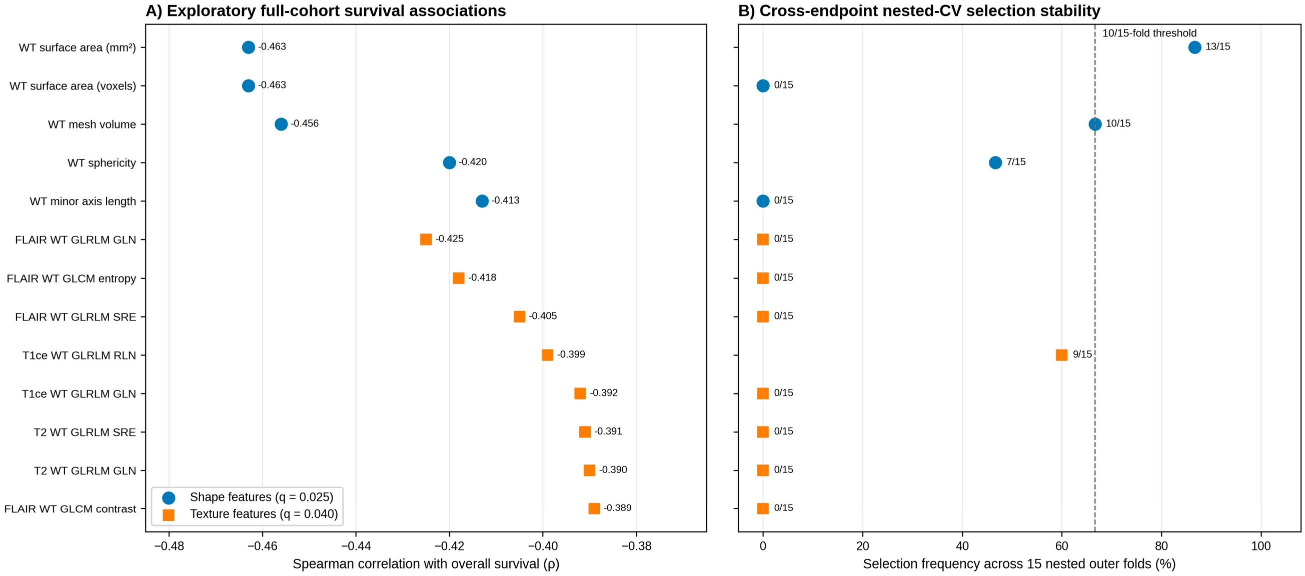

Figure S1. Tumor morphology and survival scatter plots. (A) Exploratory full-cohort Spearman correlations between the 13 FDR-significant radiomic features and overall survival. All associations were negative; the five shape features had q = 0.025 and the eight texture features had q = 0.040. (B) Cross-endpoint selection frequency of the same features across 15 nested outer folds for binary early-mortality classification. Whole-tumor surface area and mesh volume were selected in 13/15 and 10/15 folds, respectively; nine of 13 features were never selected. Because continuous overall survival and binary early mortality are different endpoints, panel B represents cross-endpoint stability rather than within-endpoint replication.

Figure S2. Distribution of AUC Values Across 15 Outer Folds

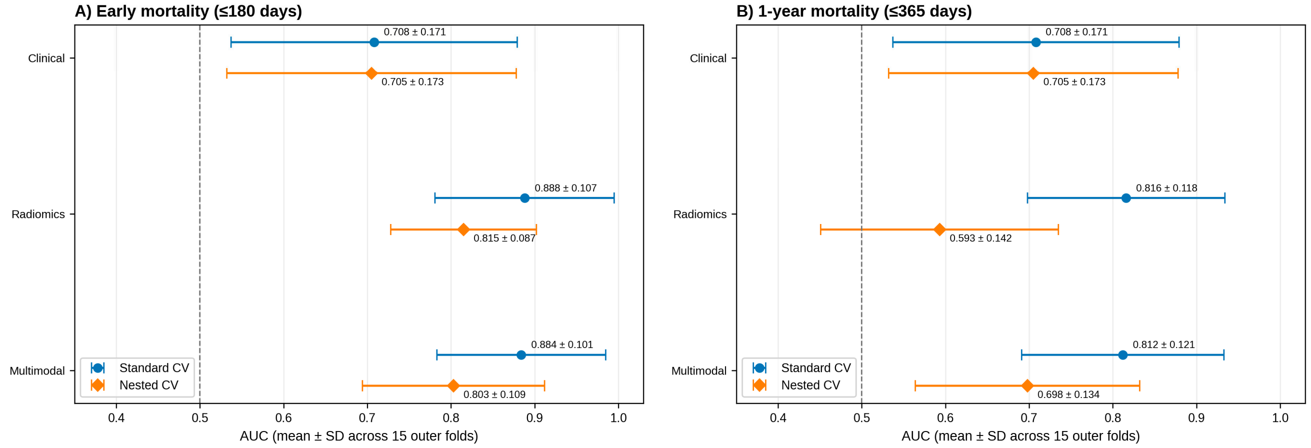

Figure S2. AUC values across the 15 outer folds (5-fold cross-validation repeated three times), summarized as mean ± SD for the standard-CV and nested-CV conditions. (A) Early mortality (≤180 days): clinical 0.708 ± 0.171 vs. 0.705 ± 0.173; radiomics 0.888 ± 0.107 vs. 0.815 ± 0.087; multimodal 0.884 ± 0.101 vs. 0.803 ± 0.109. (B) 1-year mortality (≤365 days): clinical 0.708 ± 0.171 vs. 0.705 ± 0.173; radiomics 0.816 ± 0.118 vs. 0.593 ± 0.142; multimodal 0.812 ± 0.121 vs. 0.698 ± 0.134. Error bars show the reported across-fold SD; the vertical dashed line marks chance-level discrimination (AUC = 0.5).

Table S7. Comparison of Included (n = 60) and Excluded (n = 201) Eligible Patients

From the 261 patients meeting the stated eligibility criteria, the first 60 by ascending dataset identifier constituted the analysis cohort. The descriptive comparison below is provided to characterize potential selection effects. Because individual-level data for the excluded cohort were not part of the primary analysis, no inferential claim is made from this supplementary comparison.

Variable	Included (n = 60)	Remaining eligible (n = 201)
Age, mean ± SD	59.3 ± 12.4	62.2 ± 11.8
Male, n (%)	40 (66.7%)	117 (58.2%)
MGMT methylated, n (%)	42 (70.0%)	141 (70.1%)
GTR, n (%)	26 (43.3%)	133 (66.2%)
Median OS, days (IQR)	398 (reported cohort median)	375 (203-605)
Early mortality, n (%)	16 (26.7%)	46 (22.9%)
1-year mortality, n (%)	29 (48.3%)	91 (45.3%)

Interpretation: The included cohort was broadly similar to the remaining eligible patients with respect to age, sex distribution, MGMT methylation, overall survival, and mortality rates. Gross total resection was less frequent in the included cohort (43.3% vs. 66.2%). Because the analysis cohort was a deterministic identifier-based subset rather than the full eligible population, residual selection bias cannot be excluded, particularly for EOR-related findings.
